# Human Umbilical Cord Blood-Derived CD133^+^CD34^+^ Cells Protect Retinal Endothelial Cells and Ganglion Cells in X-Irradiated Rats through Angioprotective and Neurotrophic Factors

**DOI:** 10.3389/fcell.2022.801302

**Published:** 2022-02-10

**Authors:** Siyu Chen, Minghui Li, Jianguo Sun, Dan Wang, Chuanhuang Weng, Yuxiao Zeng, Yijian Li, Shujia Huo, Xiaona Huang, Shiying Li, Ting Zou, Haiwei Xu

**Affiliations:** ^1^ Southwest Hospital/Southwest Eye Hospital, Third Military Medical University (Army Medical University), Chongqing, China; ^2^ Key Lab of Visual Damage and Regeneration and Restoration of Chongqing, Chongqing, China; ^3^ Cancer Institute, Xinqiao Hospital, Third Military Medical University (Army Medical University), Chongqing, China; ^4^ Department of Obstetrics and Gynecology, Southwest Hospital, Third Military Medical University (Army Medical University), Chongqing, China

**Keywords:** radiation retinopathy, CD133^+^CD34^+^ cells, cell transplantation, endothelial cells, retinal ganglion cells

## Abstract

Radiation retinopathy (RR) is a common complication following radiation therapy of globe, head, and neck malignancies, and is characterized by microangiopathy, neuroretinopathy, and the irreversible loss of visual function. To date, there is no effective treatment for RR. Stem cells have been clinically used to treat retinal degeneration. CD133^+^CD34^+^ cells from human umbilical cord blood (*hUCB*-CD133^+^CD34^+^ cells), a subpopulation of hematopoietic stem cells, were applied to determine their protective efficacy on irradiated rat retinas. After X-ray irradiation on the retinas, rats were intravitreally injected with *hUCB*-CD133^+^CD34^+^ cells. Transplantation of *hUCB*-CD133^+^CD34^+^ cells prevented retinal dysfunction 2 weeks post-operation and lasted at least 8 weeks. CD133^+^CD34^+^ cells were distributed along the retinal vessel and migrated to the ganglion cell layer. Moreover, grafted CD133^+^CD34^+^ cells reduced the apoptosis of endothelial and ganglion cells in irradiated rats and increased the number of survived CD31^+^ retinal endothelial cells and Brn3a^+^ ganglion cells at 2 and 4 weeks, respectively, post-operation. Co-culturing of CD133^+^CD34^+^ cells or supernatants with irradiated human retinal microvascular endothelial cells (hRECs) *in vitro,* confirmed that CD133^+^CD34^+^ cells ameliorated hREC apoptosis caused by irradiation. Mechanistically, we found that angioprotective mediators and neurotrophic factors were secreted by CD133^+^CD34^+^ cells, which might attenuate irradiation-induced injury of retinal endothelial cells and ganglion cells. hUCB-CD133^+^CD34^+^ cell transplantation, as a novel treatment, protects retinal endothelial and ganglion cells of X-irradiated rat retinas, possibly through angioprotective and neurotrophic factors.

## Introduction

Radiotherapy has been used to treat malignancies involving the globe, orbit, head, and neck, and radiotherapy usually causes several secondary complications, including radiation keratopathy, radiation iris neovascularization, neovascular glaucoma, radiation cataracts, radiation optic neuropathy, and radiation retinopathy (RR) (Reichstein 2015; Rose et al., 2018; Ramos et al., 2019). Among them, RR is characterized by progressive ischemic and proliferative changes that are similar to the development of diabetic retinopathy and can lead to irreversible loss of visual function (Rose et al., 2018). However, the pathophysiology and cellular mechanisms contributing to RR are different. RR is a chronic and progressive condition that may result from microangiopathy of the retinal vasculature after radiation exposure (Spielberg et al., 2013; Wilkinson et al., 2017). Furthermore, large retinal vessel occlusion, extensive ischemic retinopathy and maculopathy, and consequent retinal and ocular neovascularization can lead to retinal dysfunction and degeneration (Spielberg et al., 2013). The incidence rate of RR is based on the total dose of radiation, pre-existing comorbidities (e.g., diabetes mellitus, hypertension), and radiation sensitizer exposure (e.g., chemotherapy) (Horgan et al., 2010; Ferguson et al., 2017; Wilkinson et al., 2017). X-rays are the most common type of ionizing radiation that causes RR, which has an onset typically occurring between 6 months to 3 years after exposure.

To date, there is still no curative treatment for retinal pathologies related to exposure to radiotherapy. Established treatments for RR have usually been based on those for diabetic retinopathy or other ischemic retinopathies, such as laser photocoagulation, photodynamic therapy, corticosteroids, and anti-VEGF agents (Gupta and Muecke 2008; Giuliari et al., 2011; Reichstein 2015). Although treatment trials have been successfully conducted that minimize the complications, they have failed to address the pathological events caused by radiotherapy. In recent years, stem cell therapies have been proposed as a treatment option for degenerative diseases and disorders, including myocardial infarction, vascular diseases, motor neuron diseases, and neurodegenerative diseases (Sharma et al., 2012). Our previous clinical trial showed that mesenchymal stem cells are potentially safe and effective in treating degenerated retinas (Gu et al., 2018; Zhao et al., 2020). Intriguingly, stem cell-based therapeutic approaches have also received considerable attention as potential treatments for radiation-induced central nervous system damage after radiotherapy (Soria et al., 2019; Barazzuol et al., 2020). Irradiated rats engrafted with human neural stem cells (hNSCs) showed significant improvement in cognitive function than irradiated, sham-engrafted rats and behaved indistinguishably from unirradiated controls, suggesting that hNSCs are promising for functionally restoring cognition in irradiated animals (Acharya et al., 2011). Similarly, human mesenchymal stem cells were found to promote radiation-induced brain injury repair, improving neurological function in mice (Soria et al., 2019).

Umbilical cord blood (UCB) has been identified as a good source of hematopoietic stem cells (HSCs), which are identified by their capacity to self-renew and their ability to differentiate into all blood cells types (Jaing 2014; Li et al., 2019). Numerous studies have proposed many sets of cell-surface antigens to identify HSCs, such as CD133 and CD34. Human UCB (*hUCB*)-derived CD34^+^ cells have been successfully used in cell therapies for peripheral and cardiac ischemia diseases to provide vascular regeneration and proangiogenic potential (Kanji et al., 2014; Fadini et al., 2017). More recently, preclinical studies have shown that human CD34^+^ cells rapidly home into the damaged retinal vasculature in diabetic retinopathy or retinal acute ischemia-reperfusion injury following intravitreal injection and can repair retinal damage (Caballero et al., 2007; Park et al., 2012; Yazdanyar et al., 2020). CD133^+^ has been described as a more restricted subset than the CD34^+^ population, containing primitive hematopoietic stem cells (Charrier et al., 2002; Rong et al., 2018). CD133^+^ cells possess an extensive capacity for self-renewal, proliferation, and multilinear differentiation potency. A previous study showed that human CD133^+^ cells can home to an injured retinal pigment epithelium (RPE) layer, differentiate into cells with significant RPE morphology, and provide therapeutic functional recovery of the visual cycle (Harris et al., 2009). Our previous study demonstrated that the transplantation of mouse bone marrow-derived CD133^+^ stem cells could ameliorate visual dysfunction of diabetic mice (Rong et al., 2018). In addition, a subset of transplanted CD133^+^ cells migrated into the inner retina and protected retinal ganglion cells (RGCs) and rod-on bipolar cells from degeneration. However, the protective effect of hUCB-derived CD133^+^CD34^+^ cells on irradiated retinas has not been investigated.

To the best of our knowledge, this is the first study to investigate the protective effects of hUCB-derived CD133^+^CD34^+^ cells on X-irradiation-induced retinal damage in a rat model. Our results demonstrated that transplanted hUCB-CD133^+^CD34^+^ cells reduced apoptosis and ameliorated the radiation-induced dysfunction of retinal endothelial and ganglion cells through angioprotective and neurotrophic factors.

## Materials and Methods

### Animal X-Ray Irradiation

Eight-week-old Long Evens (LE) rats were purchased from the Experimental Animal Center of Third Military Medical University (Army Medical University) and raised in a specific pathogen-free room in the Animal Care Center of Southwest Hospital. All animal experiments in this study were carried out in strict accordance with the guidelines approved by the Laboratory Animal Welfare and Ethics Committee of Third Military Medical University (Army Medical University). Eight-week-old rats were randomly assigned. Rats were anesthetized via intraperitoneal injection of 1% pentobarbital sodium (200 μL/100 g). A total dose of 20 Gy X-ray in two fractions (2 × 10 Gy, with an interval of 7 days) was applied to restrictively irradiate the head area of rats through the beam. The irradiation parameters referred to the previous studies (Archer et al., 1991; Amoaku et al., 1992; Archambeau et al., 2000; Özer et al., 2017), and were as follows: SSD was 100, irradiation depth was 1.7 cm, irradiation width was 5 cm, the dose rate was 100 mV/min, the jump number was 1,000 mu, and X:Y was 35:5.

### Cell Line and Irradiation

The human retinal microvascular endothelial cell hREC line was cultured in the RPMI 1640 (Gibco, 12633012) containing 10% FBS (Gibco, 10099133C), and passage 6 (P6)-P10 generation cells were used in the experiments. hRECs were exposed to X-ray irradiation with total doses of 0.5, 1, or 2 Gy respectively for 24 h then applied for the experiments.

### Isolation and Expansion of Human Cord Blood-Derived CD133^+^CD34^+^ Stem Cells

hUCB samples (60–80 ml/unit) were obtained from healthy full-term births with parental informed consent at the Southwest Hospital, Third Military Medical University (Army Medical University). Fresh umbilical cord blood cells were separated within 12 h and used in subsequent experiments. Human mononuclear cells were isolated by density-gradient centrifugation with Ficoll-Paque PREMIUM 1.077 g/ml (GE Healthcare, Little Chalfont, United Kingdom). Red blood cells were removed with red cell lysate. According to the manufacturer’s instructions, CD133^+^ cells were isolated from human monocyte cells with a human-CD133 MicroBead Kit (130-100-830, Miltenyi Biotec) by magnetic bead separation. To assess the sorting, CD133^+^ cells were stained by primary PE-conjugated CD133 antibody (130-113-670, Miltenyi Biotec). The cell purity was 95%. CD133^+^ cells were seeded at 2 × 10^5^ cells/well in 24-well plates (NEST, China) and then cultured in StemMACS™ HSC Expansion Media, human (130-100-463, Miltenyi Biotec) at 37°C in a humidified atmosphere containing 5% CO_2_ for 7 days, then PE-conjugated anti-human-CD133 (130-113-670, Miltenyi Biotec) and APC-conjugated anti-human-CD34 (130-113-176, Miltenyi Biotec) antibodies were used to obtain CD133^+^CD34^+^ cells by flow sorting.

### CD133^+^CD34^+^ Cell Labeling and Transplantation

First, 20 μM DiI dye (Invitrogen, D3911) was configured and fully mixed. Then, the staining solution and cells of the same volume were mixed evenly and dyed at 37 °C for 30 min with oscillation. The labeled cells were washed three times with sterile HBSS (HyClone). The CD133^+^CD34^+^ cells were resuspended in sterile HBSS (HyClone) supplemented with DNase I (0.005%, Roche) for transplantation. CD133^+^CD34^+^ cell treatment was initiated the day after irradiation. Briefly, rats were given drinking water with cyclosporine A (210 mg/L, Sandoz UK, Camberley) 24 h in advance. One eye of LE rat was transplanted with CD133^+^CD34^+^cells, and the other eye was injected with HBSS (HyClone) as a sham control. A total of 2 × 10^5^ cells in 2 μL of HBSS were slowly injected into the vitreous cavity using a 33-gauge Hamilton needle (Hamilton). All animals continued to receive cyclosporine A (210 mg/L, Sandoz Camberley) in drinking water for 14 days after transplantation.

### Electroretinogram Recording

Rats were anesthetized by intraperitoneal injection of 1% pentobarbital sodium (200 μL/100 g) and Su-Mian-Xin (1 μL/100 g) after adapting to a dark environment for 12 h. Pupils were dilated with 1% tropicamide. A metal electrode was placed on each cornea as a recording electrode. The reference electrode and the grounding electrode were placed subcutaneously in the mouth and tail, respectively. According to the international electrophysiological standard, the rats were stimulated with 0.5 log (CD × s/m^2^) light. The wave was recorded and processed by the RETIport system (Purec, Japan). When the flash intensity was 0.5 log (CD × s/m^2^, 0 dB), the OP response was recorded with a 70–300 Hz band-pass filter. All the operations were carried out in a dark room with dim red safety lights. The raw data were put in Excel software to produce wave figures.

### Immunofluorescence

Rats were euthanized and the eyeballs were removed and fixed in 4% paraformaldehyde for 30 min. Under a microscope, the front sections of eyeballs were moved. Next, the eyeballs were fixed at 4°C for 2 h, then transferred to 30% sucrose for dehydration overnight at 4°C. Eyeballs were then air-dried and embedded with OCT frozen embedding agent and refrigerated at -80°C for later use. OCT of frozen sections was removed with PBS and sealed with sealant containing 0.3% Trion-X and 5% BSA, and primary antibodies were incubated overnight at 4°C then washed with 0.1% Triton-X in PBS three times at room temperature for 5 min/time. Secondary antibodies were added for 1 h at 37°C, then washed three times with PBS at room temperature for 5 min/time. DAPI was added for 10 min then washed three times with PBS at room temperature for 5 min before photography. Alexa Fluor™ 568-conjugated GS-IB4 (Invitrogen, I21412) or Alexa Fluor™ 488-conjugated GS-IB4 (Invitrogen, I21411) were incubated overnight at 4°C to label vascular network, then washed in PBS three times at room temperature for 3 min/time.

Cell slides were washed twice with PBS, 5 min each time, and 4% paraformaldehyde was added for fixation at 4°C for 15 min, then incubated in 0.3% Triton-X and 5% BSA in PBS for 15 min at room temperature. Primary antibody was added and incubated overnight at 4°C, then washed with 0.1% Triton-X in PBS at room temperature for 5 min. Secondary antibody was added and incubated at 37°C for 1 h, DAPI was added, and PBS was used for three washes at room temperature, 5 min each time. Samples were then photographed.

The primary antibodies were applied including PKCα (Abcam, ab11723, 1:300), Caspase 3 (CTS, #9661, 1:500), CD31 (Abcam, ab222783, 1:500), Cone Arrestin (Millipore, AB15282, 1:500) and Brn-3a (Santa cruz, SC8429, 1:200). Tunel was detected through *In Situ* Cell Death Detection Kit, Fluorescein or TMR red (Roche) following the kit instructions.

### Tube Formation Assay

According to previous reports, we evaluated the tube formation ability of endothelial cells. In brief, matrigel (Corning, 356277) was dissolved at 4°C 24 h in advance, and 10 μL matrigel was added to a dish (Ibidi, 81501). After being evenly spread, the matrigel was cured at 37°C for 30 min. Cells were digested and centrifuged normally. After being resuspended in a complete medium, the cells were counted. In 50 μL, 8 × 10^4^ cells/well were added to the cured matrigel, and the mixture was mixed and placed at 37°C to observe vascular tube formation.

### Western Blot Analysis

According to previous reports, proteins were detected in retinal tissue. In brief, whole retina tissues were lysed in RIPA buffer (Beyotime, P0013B) and mixed well. PMSF (Beyotime, ST505) was added before use so that the final concentration was 1 mM. After cell disruption, lysates were centrifuged at 10,000-14,000 g for 10 min. From the supernatant, protein concentration was determined using a Protein Concentration Detection Kit (Beyotime, P0006C). A total of 50 μg protein was loaded onto 6–10% SDS-PAGE gels (120 V electrophoresis for 100 min). Proteins were then transferred from gels to 0.45 μm PVDF membranes (Millipore, IPVH00010) under a constant flow of 250 mA for 2 h. PVDF membranes were sealed with 5% BSA and incubated with primary antibodies (CD31, Abcam, ab222783, 1:200; Brn-3a, Santa Cruz, SC8429, 1:100; β-Actin, CWBIO, CW0096, 1:1,000) at 4°C overnight. Then they were washed with TBST three times, and corresponding secondary antibodies were added and incubated at 37°C for 1 h, washed with TBST three times, and exposed by an ECL Chemiluminescence Kit (Thermo, 32209).

### Protein Array

The protein array was detected through Proteome Profiler Human Angiogenesis Array (R&D, ARY007). A total of 2.0 ml of array buffer 7 was added to the reaction plate and the reaction film was placed in it until the blue dye on the film disappeared, then incubated on a shaker for 1 h. While the membranes are blocking, prepare samples by adding up to 1.0 ml of each sample to 0.5 ml of Array Buffer 4 in separate tubes. Adjust to a final volume of 1.5 ml with Array Buffer 5 as necessary. A recombinant detection antibody factor (15 μL) was added to each sample, mixed well, and incubated at room temperature for 1 h. Array buffer 7 was removed and the sample/antibody mixture was added. Samples were covered and incubated overnight on a shaker at 2–8°C. The membrane was removed and placed in 1× of wash buffer in a single plastic container of 20 ml for 10 min and repeated twice. A total of 2.0 ml of streptavidin-HRP diluted with buffer five was added into each well, covered with a lid, and incubated on a shaker at room temperature for 30 min. Membranes were washed for 10 min three times, then drained. A total of 1.0 ml of the prepared chemical reagent mixture was evenly dropped on each membrane and incubated for 1 min at room temperature before radiological automatic imaging of the membrane.

### Statistical Analysis

Data are expressed as means ± SEM from independent experiments. For comparison between two groups, the significance of differences between groups was evaluated by independent samples *t*-test. For comparison between multiple groups, the significance of differences between groups was evaluated by a one-way analysis of variance. * *p* < 0.05 was considered significant, and ** *p* < 0.01 and *** *p* < 0.001 were considered extremely significant. Graphs were plotted and analyzed using GraphPad Prism version 6.0 (GraphPad Software, La Jolla, CA, United States).

## Results

### Loss of Retinal Vascular Endothelial Cells and Ganglion Cells in Retinal Irradiated-Rats

**FIGURE 1:**
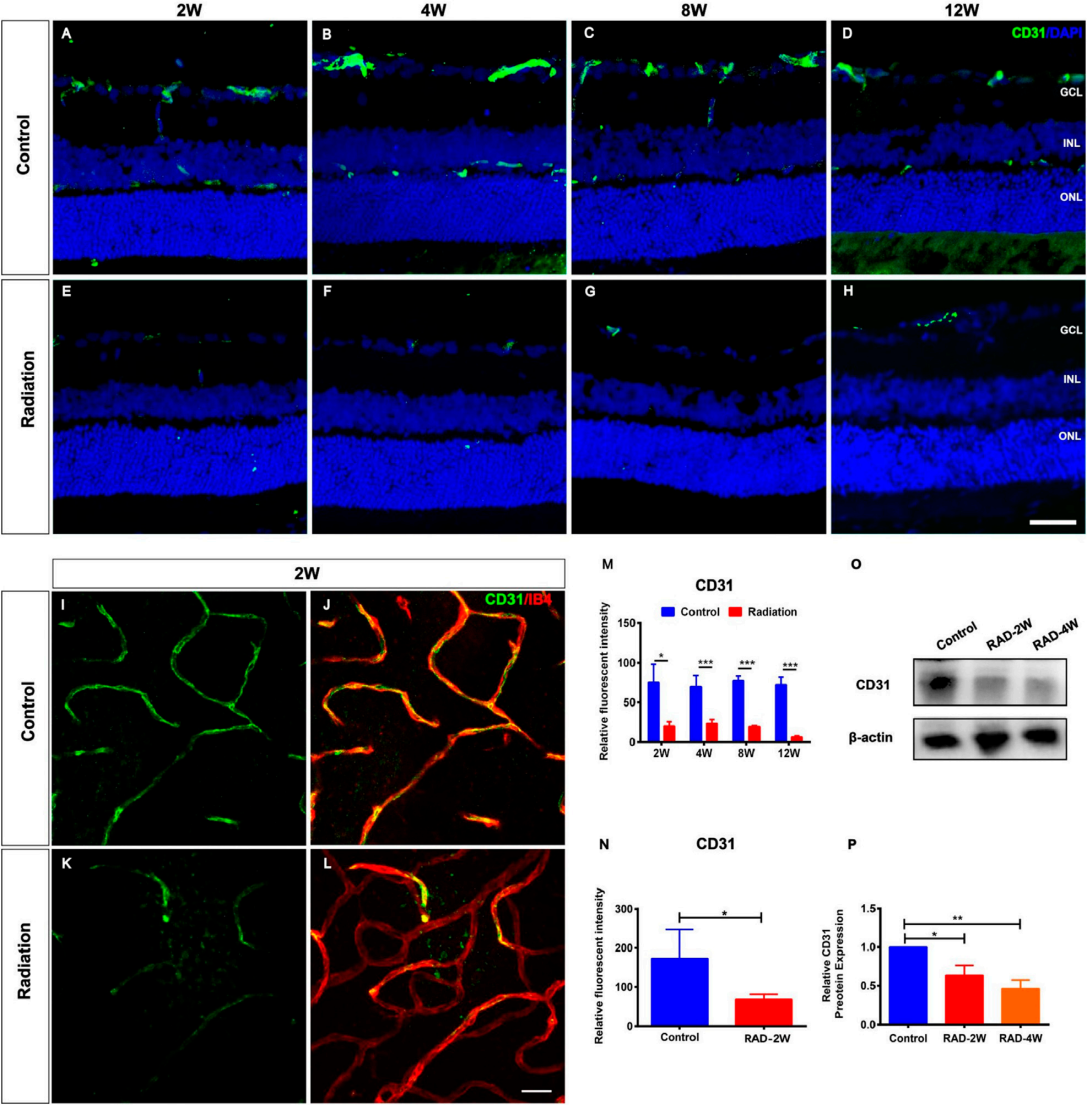
Spatiotemporal changes of CD31^+^ endothelial cells in the irradiated LE rats. **(A–H)**: Representative images of CD31^+^ (Green) retinal endothelial cells in control and irradiated rat retina after 2, 4, 8, and 12 weeks radiation. Scale bar: 50 μm. **(I–L)**: The retinal blood vessels showed by CD31 and IB4 staining in the wholemount retina. Scale bar: 50 μm. **(M)**: The fluorescence intensity statistics of CD31 in retinal sections. **(N)**: The fluorescence intensity statistics of CD31 of retinal blood vessels in the wholemount retina. **(O)**: WB analysis of the protein expression of CD31 in the retina. **(P)**: The statistics of WB grayscale of CD31 protein expression. N = 3. Data are depicted as means ± SEM. (* *p* < 0.05, *** *p* < 0.001). GCL: ganglion cell layer; INL: inner nuclear layer; ONL: outer nuclear layer.

To characterize the impact of radiation on rat retinas, eight-week-old LE rats were subjected to eye X-ray irradiation (total dose 20 Gy). Compared with controls, the histopathological analysis of rat retina tissue sections by fluorescence microscopy revealed the loss of CD31^+^ retinal vascular endothelial cells from 2 to 12 weeks post-irradiation (Figures 1A–H). The relative fluorescent intensity of CD31 expression in the retina was significantly decreased from 2 to 12 weeks post-radiation (Figure 1M). Moreover, CD31^+^ retinal vascular endothelial cells were also damaged in retina flat mounts with a reduction of fluorescent intensity of CD31 at 2 weeks post-irradiation (Figures1I–L,N). Western blot (WB) analysis showed that the protein level of CD31 was significantly reduced after irradiation treatment (Figures 1O,P). This observation was not in line with the CD31^+^ vascular endothelial cells in flat mounts of the choroid (Supplementary Figure S1), confirming the specific loss of CD31^+^ retinal vascular endothelial cells in irradiated retinas.

**FIGURE 2:**
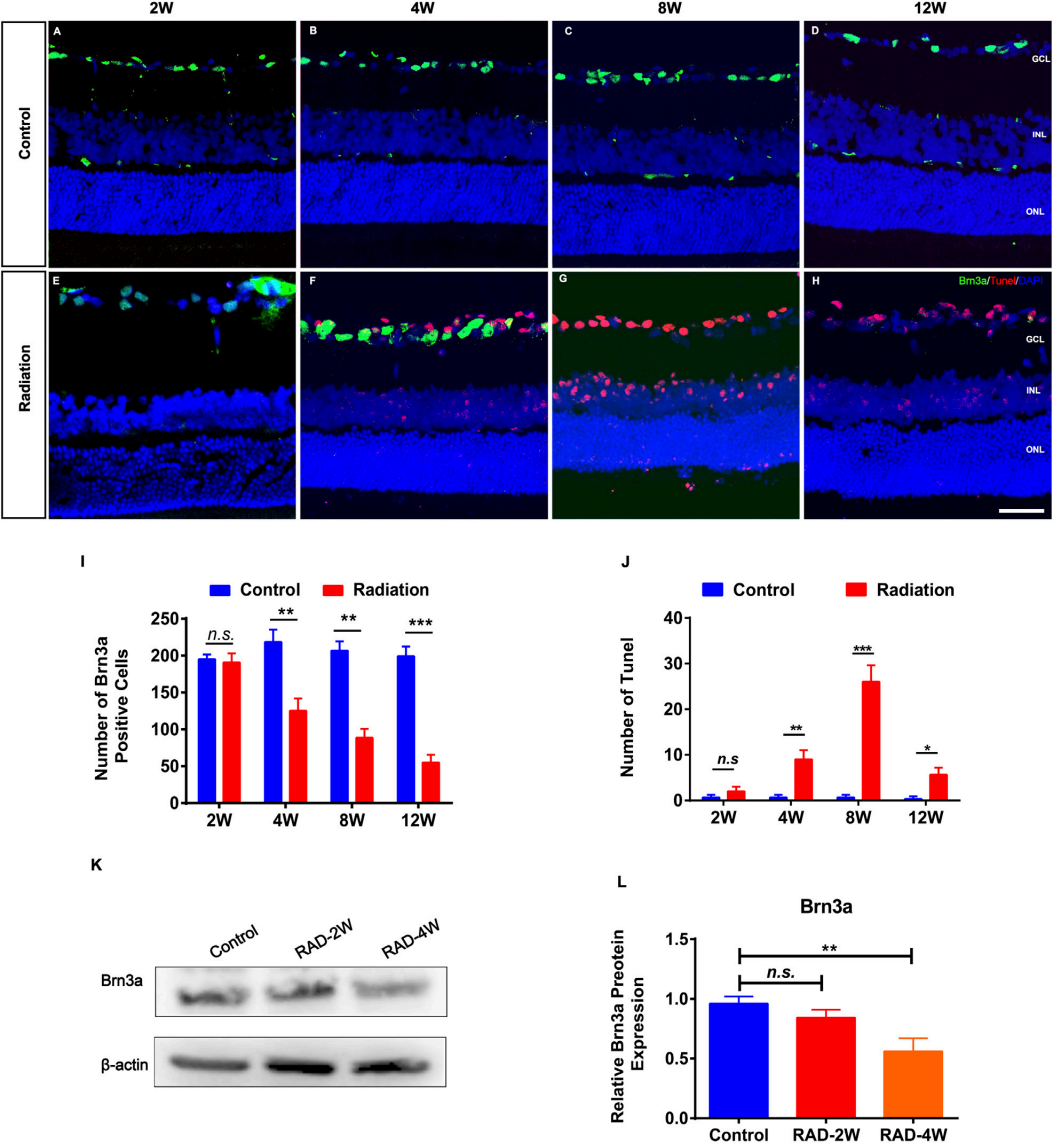
The apoptosis of retinal ganglion cells in irradiated LE rats. **(A–H)**: Representative images of Brn3a^+^ ganglion cells (Green) and apoptotic cells (Red) in control and irradiated rat retina after 2, 4, 8, and 12 weeks radiation. Scale bar: 50 μm. **(I,J)**: The number of Brn3a^+^ and Tunel positive cells in the retina. **(K)**: WB analysis of the protein expression of Brn3a in the retina. **(L)**: The statistics of WB grayscale of Brn3a protein expression. N = 3. Data are depicted as means ± SEM. (* *p* < 0.05, ** *p* < 0.01, *** *p* < 0.001). GCL: ganglion cell layer; INL: inner nuclear layer; ONL: outer nuclear layer.

RGC staining of Brn3a in irradiated retinas showed significant loss of ganglion cells after 4 weeks but not 2 weeks (Figures 2A–I). This was consistent with the TUNEL staining showing that the apoptosis of RGCs was significantly increased at 4 weeks after radiation, and particularly at 8 weeks and up to 12 weeks (Figures 2A–H,J). In addition, WB confirmed that Brn3a protein was significantly decreased in irradiated rat retinas 4 weeks later (Figures 2K,L). Moreover, fluorescence microscopy showed that the numbers of PKCα^+^ bipolar cells and Arrestin^+^ cone photoreceptors were reduced by irradiation (Supplementary Figures S2, S3). These results suggested secondary damage of retinal neurons after irradiation.

### Transplanted hUCB-CD133^+^CD34^+^ Cells Improved the Visual Function of Irradiated Rats

**FIGURE 3:**
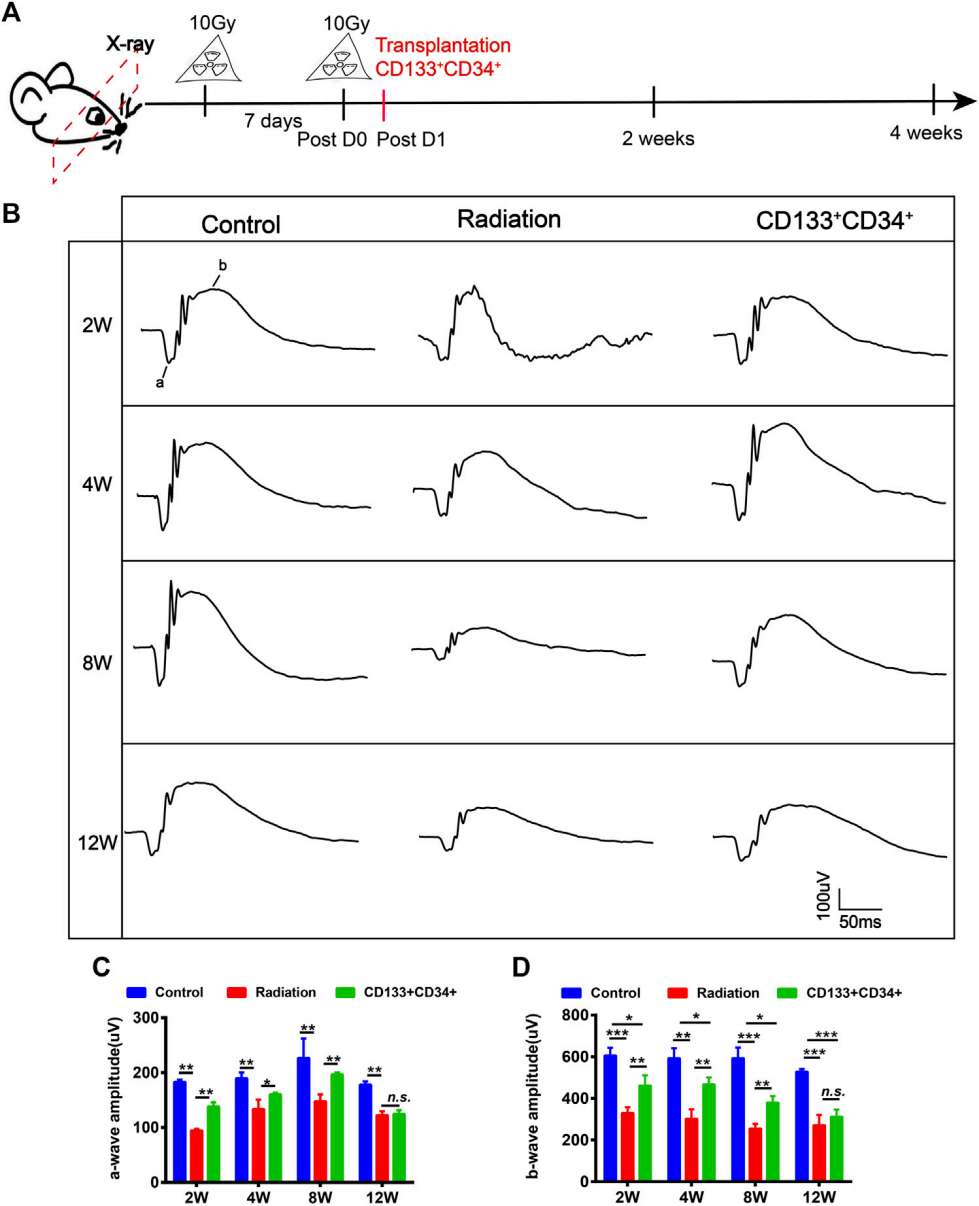
Transplanted CD133^+^CD34^+^ cells improved retinal dysfunction of irradiated LE rats. **(A)**: The schematic diagram of design for irradiated rat models and cell transplantation experiment. **(B)**: The a- and b-wave amplitude of ERG from control, irradiated, and transplanted group. **(C,D)**: The statistics of a- and b-waves amplitude on control, irradiated, and cell transplanted group from 2 to 12 weeks. N = 3. Data are depicted as means ± SEM. (* *p* < 0.05, ** *p* < 0.01, *** *p* < 0.001).

Fluorescent cell sorting was used to obtain CD133^+^CD34^+^ cells from hUCB (Supplementary Figure S4). CD133^+^CD34^+^ cells expressed the endothelial marker CD31 and the neuronal marker βIII-tubulin (Tuj1) (Supplementary Figure S4C,D) after inducing differentiation, demonstrating the ability of CD133^+^CD34^+^ cells to differentiate into endothelial cells and neurons. *hUCB*-CD133^+^CD34^+^ cells were intravitreally transplanted into irradiated rats at the next day post-irradiation (Figure 3A), and we examined whether they could ameliorate retinal injury (Figure 3B). Electroretinogram (ERG) a-, and b-waves were recorded to evaluate the retinal function (Figures 3B–D). A significant difference was observed in the a- and b-wave amplitude between the irradiation and control groups, with retinas from irradiated rats showing a decreased wave response after 20 Gy irradiation (Figures 3B–D). Transplantation of *hUCB-*CD133^+^CD34^+^ cells significantly increased the b-wave amplitude until 8 weeks, but not 12 weeks (Figures 3B,D). Similarly, increased amplitude of a-waves was also observed after CD133^+^CD34^+^ transplantation, with statistical significance from 2 to 8 weeks, but not 12 weeks (Figures 3B,C).

### Transplanted hUCB-CD133^+^CD34^+^ Cells Ameliorated the Retinal Vascular Structure and Function of Irradiated Rats

**FIGURE 4:**
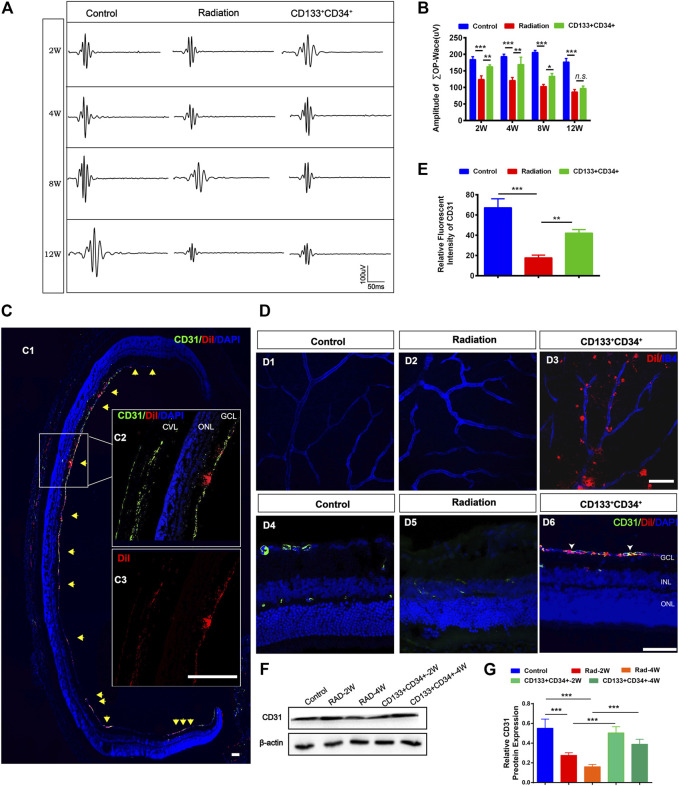
Transplanted CD133^+^CD34^+^ cells protect retinal vasculature injuries after radiation. **(A)**: Representative images of OPs waves of fERG between control, irradiated, and cell transplanted group. **(B)**: The statistics of ∑OPs wave amplitude from 2 to 12 weeks. **(C)**: Intravitreous transplantation of CD133^+^CD34^+^ cells in X-irradiated rats. C1: The distribution of the transplanted CD133^+^CD34^+^ cells in retinal section *Discussion* week-transplantation later; Scale bar: 50 μm. C2: The magnification of the retinal section. C3: Individual channel of Dil labeled CD133^+^CD34^+^ cells. Scale bar: 50 μm. **(D)**: D1-D6: The distribution of CD133^+^CD34^+^ cells in the wholemount retina and retinal section after 4-week transplantation. **(E)**: CD31 fluorescence intensity statistics at 4 weeks. **(F)**: WB analysis of the CD31 protein expression after transplantation at 2 and 4 weeks. **(G)**: The statistics of the relative CD31 protein expression in the rat retina. N = 3. Data are depicted as means ± SEM. (* *p* < 0.05, ** *p* < 0.01, *** *p* < 0.001, ANOVA-test). GCL: ganglion cell layer; INL: inner nuclear layer; ONL: outer nuclear layer; CVL: choroidal vascular layer.

The oscillating potential (OP) is sensitive to vascular disturbances in the retina (Lovasik and Kergoat 1991). Transplantation of CD133^+^CD34^+^ cells significantly increased the ∑OPs from 2 to 8 weeks, but not at 12 weeks, post-irradiation (Figures 4A,B). Thus, we identified the effects of transplanted CD133^+^CD34^+^ cells on the retinal vessel. Before cell transplantation, CD133^+^CD34^+^ cells were labeled with DiI to better trace CD133^+^CD34^+^ cells in the retina. CD133^+^CD34^+^ cells were found mainly located in the ganglion cell layer at post-operational 4 weeks (Figure 4C) and distributed along with the retinal vessel network (Figure 4D). A significantly increased CD31 fluorescence intensity at post-operational 4 weeks was detected compared with the control (Figures 4D,E). Moreover, the protein level of CD31 significantly increased after CD133^+^CD34^+^ cell transplantation at 2 and 4 weeks (Figures 4F,G), indicating that transplanted CD133^+^CD34^+^ cells ameliorated endothelial cell injuries caused by irradiation, leading to a potential repair of retinal vascular function.

### Transplanted hUCB-CD133^+^CD34^+^ Cells Protect Retinal Ganglion Cells in Retinal-Irradiated Rats

**FIGURE 5:**
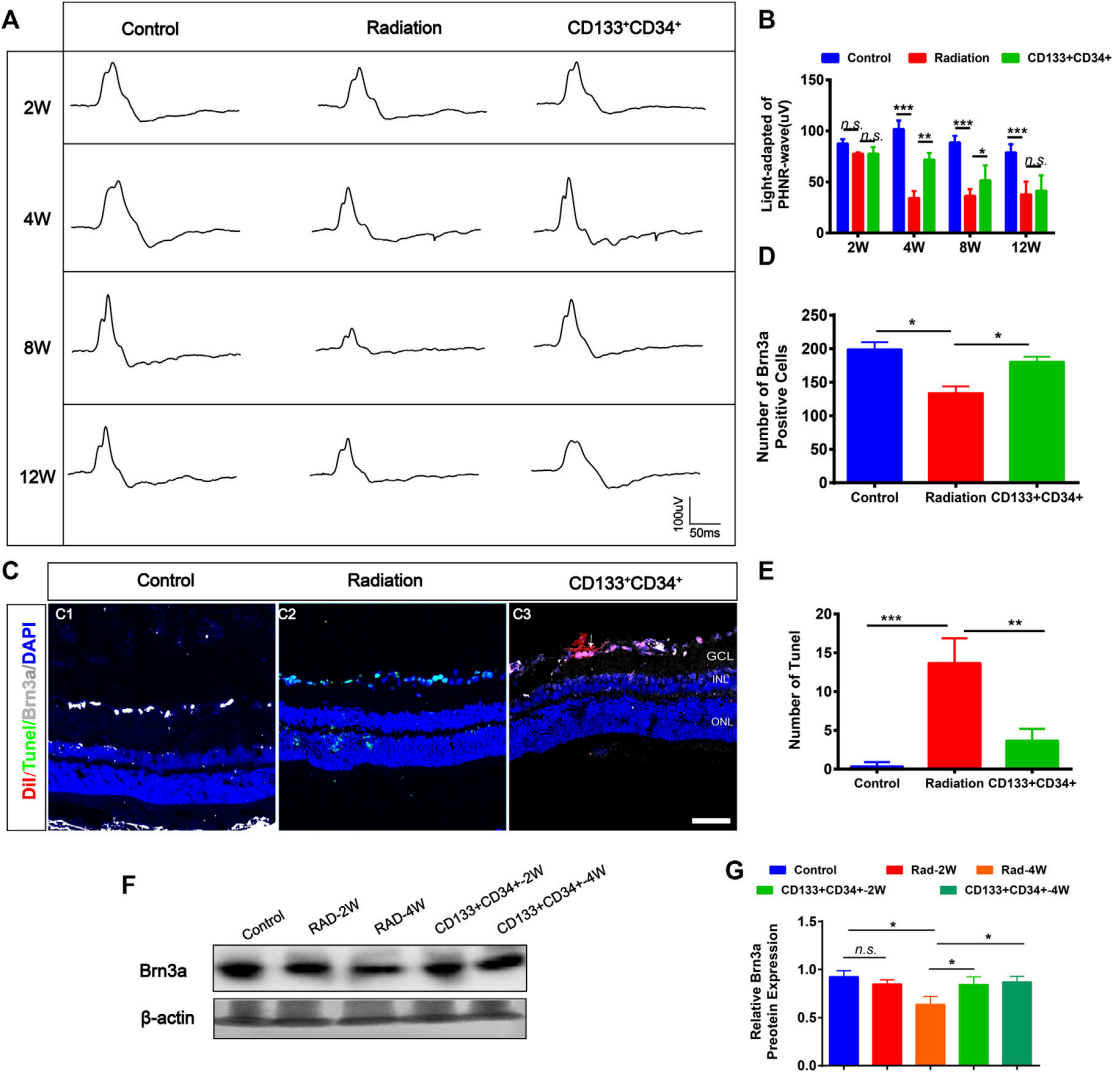
Transplanted CD133^+^CD34^+^ cells rescued radiation injured RGCs of LE rats. **(A)**: Representative images of light-adapted of PHNR-wave on control, irradiated, and transplanted group after transplantation 2, 4, 8, and 12 weeks. **(B)**: The statistics of PHNR-wave on four timepoints. **(C)**: Representative images of Brn3a^+^ ganglion cells (Gray) and Tunel (Green) staining on the retina from control, irradiated, and transplanted group at transplantation 4 weeks. Scale bar: 50 μm. **(D–E)**: The statistics of the numbers of Brn3a and apoptotic positive cells in the retina. **(F)**: WB analysis of the Brn3a protein expression. **(G)**: The statistics of relative Brn3a protein expression in WB analysis. N = 3. Data are depicted as means ± SEM. (* *p* < 0.05, *** *p* < 0.01, *** *p* < 0.001, ANOVA-test). GCL: ganglion cell layer; INL: inner nuclear layer; ONL: outer nuclear layer.

The photopic negative response (PHNR) was measured to provide specific information about RGC activity after transplantation (Moss et al., 2015). The PHNR-wave was significantly decreased in rat retinas at 4 weeks after irradiation (Figures 5A,B). Transplantation of cells significantly increased the light-adapted PHNR wave in irradiated rat retinas at 4 and 8 weeks after transplantation. However, there was no significant difference in PHNR waves between irradiated rat retinas and transplanted rat retinas at 12 weeks. Furthermore, the immunofluorescence staining showed that the number of Brn3a^+^ ganglion cells was significantly increased at 4 weeks after transplantation (Figures 5C,D). In addition, apoptosis was significantly decreased at 4 weeks after transplantation (Figures 5C–E). Moreover, increased expression of Brn3a protein in transplanted retinas was detected at 4 weeks (Figures 5F,G). Therefore, these results showed that transplanted CD133^+^CD34^+^ cells protected injured ganglion cells, improved their function from 4 weeks after transplantation.

### hUCB-CD133^+^CD34^+^ Cells Protected hRECs from Irradiation-Induced Injury *in vitro*

Vascular endothelial cells have been implicated as primary targets of damage in radiotherapy. The morphological changes and apoptosis of hRECs were evaluated after X-irradiation. Immunofluorescence staining showed significantly increased apoptosis according to TUNEL staining and caspase 3 after irradiation with 0.5, 1, or 2 Gy of hRECs (Supplementary Figures S5A,B). To evaluate the response of hRECs to radiation, we performed tube formation assays. Our data showed that the capillary-like networks of hRECs on matrigel were reduced in a dose-dependent manner in irradiated cells. Compared with the control group, the numbers of nodes, vessel length, and branch length were significantly decreased in cells treated with 0.5, 1, or 2 Gy irradiation 24 h later (Supplementary Figure S5C–F), suggesting that retinal vascular endothelial cells were sensitive to irradiation.

**FIGURE 6:**
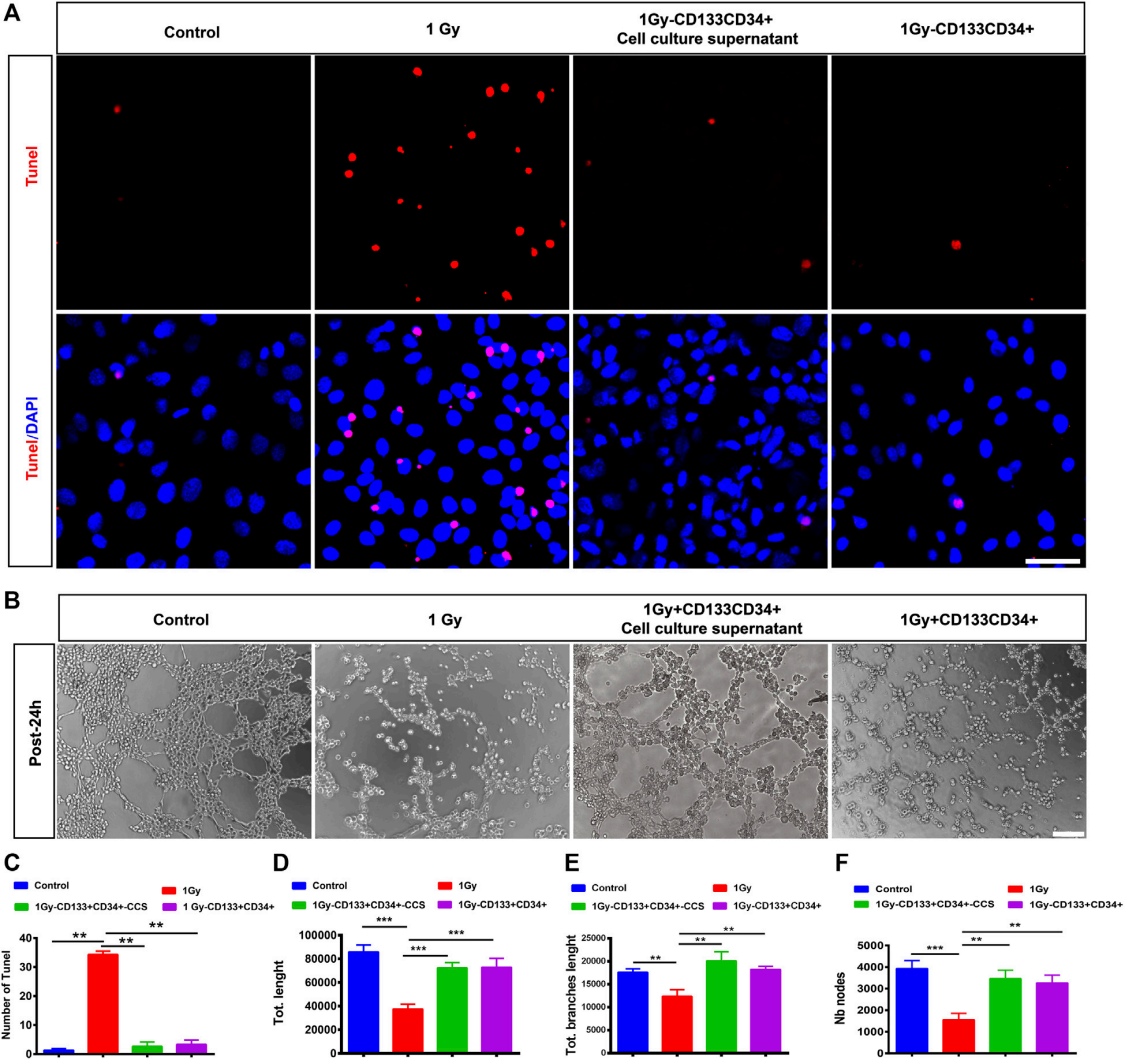
Co-cultured CD133^+^CD34^+^ cells or supernatant protected hRECs from radiation-induced injuries *in vitro.* **(A)**: Representative images showing the expression of Tunel on hRECs with control (0 Gy), irradiated (1 Gy), coculturing with CD133^+^CD34^+^ cell and CD133^+^CD34^+^ cell supernatant 24 h later *in vitro*. Scale bar: 50 μm. **(B)**: The tube formation assay of hRECs with 1 Gy X-ray irradiation or irradiation combined coculturing with CD133^+^CD34^+^ cells and CD133^+^CD34^+^ cell supernatants 24 h later. Scale bar: 100 μm. **(C)**: The statistics of the number of Tunel positive cells. **(D–F)**: The statistics of the number of nodes, vessel length, and branches length. N = 3. Data are depicted as means ± SEM. (** *p* < 0.01, *** *p* < 0.001, ANOVA-test).

To further delineate the effects of CD133^+^CD34^+^ cell transplantation, we performed the Transwell co-culture assays of CD133^+^CD34^+^ cells or CD133^+^CD34^+^ cell culture supernatants with irradiated hRECs. Our results showed that the apoptotic cells of 1 Gy-irradiated hRECs were decreased after co-culturing with CD133^+^CD34^+^ cells or CD133^+^CD34^+^ cell culture supernatants (Figures 6A,C). In addition, CD133^+^CD34^+^ cells or CD133^+^CD34^+^ cell culture supernatant repaired the dysfunction of hRECs caused by 1 Gy radiation as illustrated by the abnormal tube formation such as the number of nodes, vessel length, and branch length (Figures 6B,D–F). Moreover, we found that co-culturing with CD133^+^CD34^+^ cells or CD133^+^CD34^+^ cell culture supernatants also decreased 0.5 Gy X-ray-induced hRECs apoptosis and rescued the potential for tube formation (Supplementary Figure S6). These results indicated that CD133^+^CD34^+^ cell culture supernatants could ameliorate the damage caused by radiation in hRECs.

### Angioprotective Factors and Neurotrophic Factors Secreted by CD133^+^CD34^+^ Cells

**FIGURE 7:**
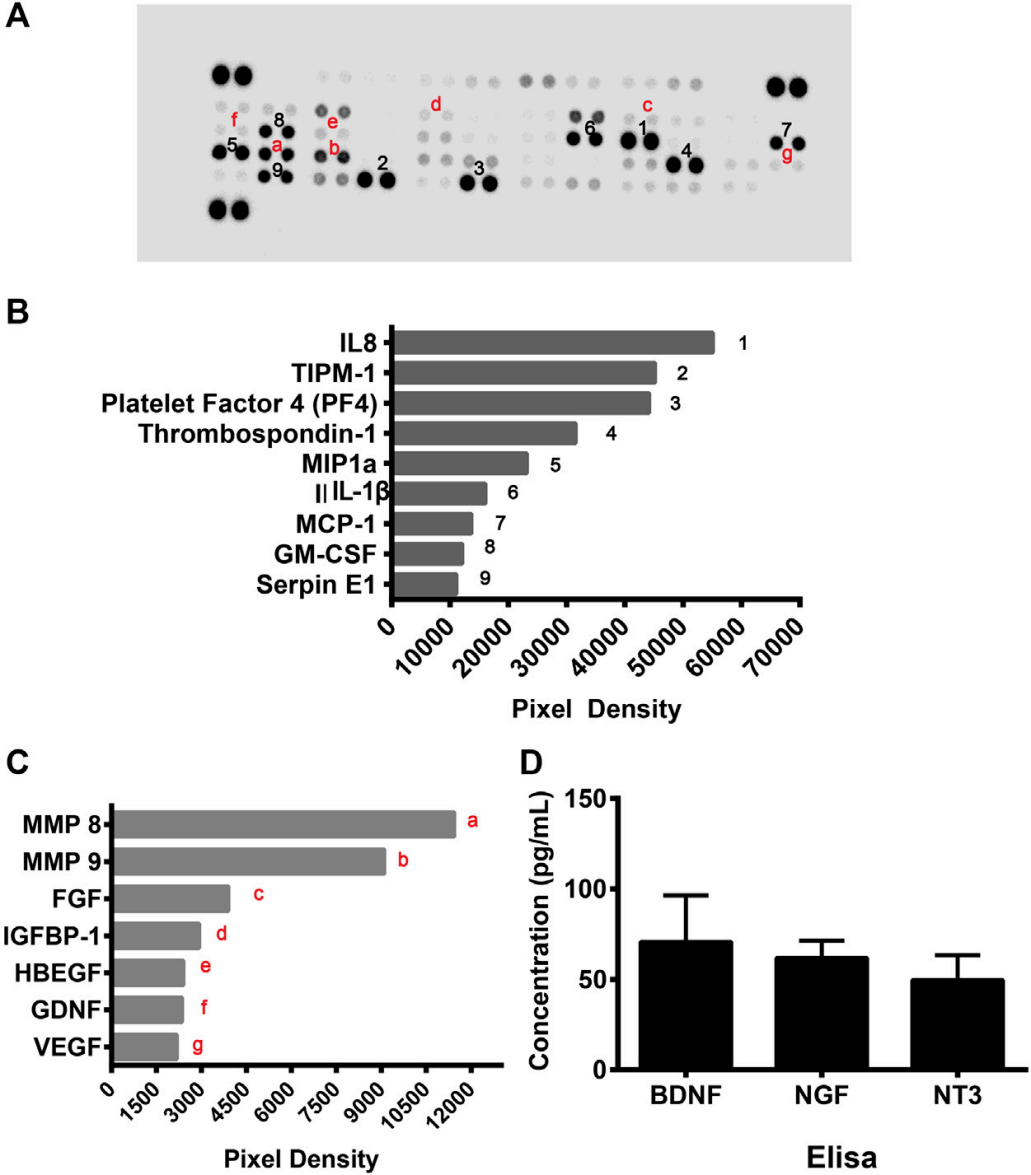
CD133^+^CD34^+^ cell supernatant mediated angioprotective and neurotrophic effect. **(A)**: Human angiogenesis protein array was used to profile angio-rassociated proteins in the CD133^+^CD34^+^ cell supernatant secretome. **(B)**: The top 9 highly expressed factors in the CD133^+^CD34^+^ cells. **(C)**: The secretome from the CD133^+^CD34^+^ cells contained angio-associated factors. **(D)**: ELISA revealed the concentration of neurotrophic factors in CD133^+^CD34^+^ cell supernatant.

As the above data showed that cell culture supernatants of CD133^+^CD34^+^ cells have a protective effect on hRECs post-radiation, we suspected that CD133^+^CD34^+^ cells exert therapeutic effects through the secretome. To further illustrate the protective mechanism of CD133^+^CD34^+^ cells, a human cytokine array was performed to profile secretory mediators in the CD133^+^CD34^+^ cell secretome (Figure 7A). It revealed that the top nine highest levels of factors in CD133^+^CD34^+^ cell supernatants were interleukin 8 (IL-8), tissue inhibitors of metalloproteinase 1 (TIPM-1), platelet factor 4 (PF4), thrombospondin-1, macrophage inflammatory protein 1α (MIP1α), interleukin 1β (IL-1β), monocyte chemoattractant protein-1 (MCP-1), granulocyte-macrophage colony-stimulating factor (GM-CSF), and Serpin E1 (Figure 7B). Among these, the most highly expressed factor, IL-8, also known as CXCL8, has anti-apoptotic properties and contributes to endothelial cell survival (Li et al., 2003; Zhang et al., 2021). Additionally, IL-8 has also been shown to promote the homing of bone marrow-derived cells to injured sites and differentiation into endothelial cells (Li et al., 2008; Hou et al., 2014). Moreover, TIMP1, which was also previously shown a role in anti-apoptosis (Lao et al., 2019; Chen et al., 2020), was the second-highest factor in CD133^+^CD34^+^ cell supernatants. Other angio-associated factors, such as matrix metalloproteinase 8 and 9 (MMP8, MMP9), fibroblast growth factor (FGF), insulin-like growth factor binding protein 1 (IGFBP-1), heparin-binding epidermal growth factor (HBEGF), glial cell line-derived neurotrophic factor (GDNF), and VEGF, were also detected with relatively lower levels of expression in CD133^+^CD34^+^ cell supernatants (Figure 7C). Enzyme-linked immunosorbent assay (ELISA) was performed to evaluate the expression of neuroprotective factors in CD133^+^CD34^+^ cell supernatants. We found the expression of brain-derived neurotrophic factor (BDNF), nerve growth factor (NGF), and neuronutrient 3 (NT3) in CD133^+^CD34^+^ cell supernatants (Figure 7D). Therefore, CD133^+^CD34^+^ cell-derived anti-apoptosis factors and neurotrophic factors could be mainly responsible for the reduction of irradiation-induced apoptosis and retinal endothelial and ganglion cell damage.

## Discussion

In this study, we investigated the protective effects of *hUCB*-CD133^+^CD34^+^ cell transplantation on X-irradiated rat retinas, and our findings strongly suggest that CD133^+^CD34^+^ cells can meliorate the injuries caused by irradiation, which might shed light for providing a new therapeutic strategy of radiation retinopathy.

RR animal model studies performed using capuchin monkey eyes irradiated with an external beam, and the first change detected around 10 months post-irradiation was the focal loss of endothelial cells and pericytes, yielding acellular capillaries (Irvine and Wood 1987; Ramos et al., 2019). Later, Hiroshiba et al. demonstrated that irradiation reduced the leukocyte velocity in capillaries and gradually increased leukocyte entrapment in the retinal microcirculation (Hiroshiba et al., 1999). The activated leukocytes and major retinal vessels that were significantly constricted 7 days after 20 Gy irradiation may participate in the pathogenesis and exacerbation of capillary closure and subsequent microvascular dysfunction in RR. In our study, we used a retinopathy dose (20 Gy) and chose a time interval that was sufficient to observe irradiation-induced retinal impairment in rat eyes. The loss of endothelial cells was consistent with previous studies and showed that the ultimate response of the retina to irradiation is vision loss as a consequence of vascular and parenchymal damage (Amoaku and Archer 1990a, b).

In addition, we studied the effects of irradiation exposure on hRECs at doses of 0.5, 1, or 2 Gy; these doses have been found to induce primary rat retinal cell impairment (Gaddini et al., 2018). hRECs showed significantly induced apoptosis with 0.5, 1, or 2 Gy irradiation as illustrated by TUNEL assays and caspase 3 stainings. These are consistent with previous studies showing that endothelial cells are particularly sensitive to irradiation (Baselet et al., 2019; Wijerathne et al., 2021). Moreover, the tube formation of hRECs was markedly attenuated with irradiation; for instance, the number of nodes, vessel length, and branch length was significantly decreased, suggesting irradiation-induced dysfunction. Retinal vascular endothelial cell injury was detected first after irradiation in the present study, which is consistent with previous reports in which the initial pathological change was retinal vascular endothelial cell injury and loss (Archer et al., 1991; Wilkinson et al., 2017). Additionally, retinal perfusion changes were found in patients with radiation-related retinopathy (Rose et al., 2018). Like retina vessels, the choroidal circulation was profoundly affected by RR, as confirmed by choroidal vascular lesions (Takahashi et al., 1998; Spaide et al., 2002). However, no significant changes were found in irradiated rat choroids in our study. The reason could lie in that RR being closely associated with the total radiation dose and the total elapsed time in the course of radiation treatment (Wilkinson et al., 2017).

RGCs were significantly reduced by irradiation after 4 weeks rather than 2 weeks. Also, apoptotic changes of RGCs were detected after irradiation for 4 weeks. Moreover, photoreceptors and bipolar cells were also damaged after 4-week. Retinal dysfunction was illustrated by decreased a-, b-, and OPs-, and the PHNR wave amplitude also decreased after irradiation. These results suggested retinal neuron damage secondary to retinal vascular endothelial cell damage after irradiation. This cascade of events was in line with previous studies showing vasculopathy leading to retinal dysfunction and degradation (Amoaku and Archer 1990a; Wilkinson et al., 2017). The subsequent damage to RGCs and other neurons and eventually retinal dysfunction may occur via irradiation-induced vascular inflammatory responses and oxidative stress (Santiago et al., 2018). Therefore, the protection of retinal endothelial cells has always been the basis of treatment strategies for RR, and currently, vascular protection strategies are being used.

HSCs can produce endothelial progenitor cells that contribute to the repair of damaged blood vessels (Slayton et al., 2007). Previous studies have shown that in induced retinal ischemia, neovascularization is prompted after durable HSC transplantation, indicating that HSCs can differentiate into all hematopoietic cell lineages and endothelial cells that revascularize adult retinas (Grant et al., 2002). More recently, interest has grown rapidly in the use of UCB as an alternate source of HSCs for transplantation (Shahaduzzaman and Willing 2012; Yu et al., 2013; Noroozi-Aghideh and Kheirandish 2019; Papa et al., 2019). *hUCB*-CD34^+^ cells present the potential of vascular regenerative and proangiogenic, can be recruited into developing vessels where they exhibit a potent paracrine proangiogenic action (Pozzoli et al., 2011). A previous study has shown that intravitreal injection of human CD34^+^ cells resulted in retinal homing and integration of these human cells with preservation of the retinal vasculature in diabetic retinopathy (Yazdanyar et al., 2020). In our previous study, CD133^+^ stem cells ameliorated the visual dysfunction of diabetic retinopathy mice (Rong et al., 2018). Interestingly, the present study is the first to investigate the radioprotective efficacy of *hUCB-*CD133^+^CD34^+^ cells on irradiated mature rat retinas. We used ERG to assess the radioprotective effects of CD133^+^CD34^+^ cells on visual function after irradiation. Compared with controls, transplantation of CD133^+^CD34^+^ cells could significantly improve the a- and b-wave amplitudes at 8 weeks, but not at 12 weeks. Similarly, transplantation of CD133^+^CD34^+^ cells could significantly increase the ∑OPs at 8 weeks, but not at 12 weeks, probably because the transplanted cells cannot survive for long. Moreover, transplanted CD133^+^CD34^+^ cells increased the PHNR-wave after 4 weeks. These results suggested that transplanted CD133^+^CD34^+^ cells can ameliorate X-ray radiation-induced retinal dysfunction.

An *in vitro* coculturing system was applied to investigate the mechanism of the radioprotective efficacy of *hUCB*-CD133^+^CD34^+^ cells. *hUCB*-CD133^+^CD34^+^ cells or cell supernatants were found to decrease irradiated hREC apoptosis and to improve the function of hRECs. Interestingly, there was no significant difference between *hUCB*-CD133^+^CD34^+^ cells or cell supernatant cocultures. Therefore, the radioprotective efficacy of *hUCB*-CD133^+^CD34^+^ cells may be mediated by small molecules or cytokines derived from cells. Subsequently, a human angiogenesis array was applied to profile pro-angiogenesis mediators in the CD133^+^CD34^+^ cell supernatant secretome. The most highly expressed factor, IL-8, is an anti-apoptosis factor and has been shown to promote endothelial cell survival (Li et al., 2003; Zhang et al., 2021). Furthermore, IL-8 contributes to the homing of bone marrow-derived cells to injured sites and the differentiation into endothelial cells (Li et al., 2008; Hou et al., 2014). Moreover, TIMP1, which was also previously shown a role in anti-apoptosis (Lao et al., 2019; Chen et al., 2020), was the second-highest factor in CD133^+^CD34^+^ cell supernatants. Therefore, the top two highly expressed factors support the radioprotective effect of CD133^+^CD34^+^ cells demonstrated in this study. Moreover, MMP8 and MMP9 were highly expressed in CD133^+^CD34^+^ cell supernatants. MMPs regulate vascular cell proliferation and apoptosis by proteolytically cleaving and modulating bioactive molecules and relevant signaling pathways (Chen et al., 2013; Wang and Khalil 2018). MMP8 and MMP9 also have been reported to function in stem/progenitor cell mobilization and recruitment in blood vessel formation and vascular remodeling (Chen et al., 2013). Therefore, angioprotective factors from CD133^+^CD34^+^ cells could mediate the repair of injured vascular endothelial cells caused by irradiation. However, in this study, VEGF was also detected in the protein array of CD133^+^CD34^+^ cell supernatants but with a much lower expression, predicting a lower risk of neovascularization.

In addition, *hUCB*-CD133^+^CD34^+^ cell neuroprotective actions in retinal tissues may be mediated by a complex cascade of neurotrophins that have been classically related to damage prevention and neuroretinal tissue repair (Martinez-Moreno et al., 2018). Several neurotrophic factors, such as BDNF, NGF, and NT3 were detected in CD133^+^CD34^+^ cell supernatants. BDNF is an essential neurotrophin that supports the function and survival of RGC, and is considered a possible treatment to prevent diabetic retinopathy-induced neuroretinal damage (Mysona et al., 2018; Suzumura et al., 2020). Thus, there is a possible protective effect of CD133^+^CD34^+^ cell-derived BDNF on irradiation-induced retinal cell damage, with specific emphasis on RGC degeneration. Administration of exogenous NGF has been shown to ameliorate retinal degeneration (Garcia et al., 2017). Thus, the expression of NGF in retinal tissue after transplantation may be an attempt by CD133^+^CD34^+^ cells to protect the retina from RGC degeneration. NT3 also played important roles in neuron survival and retinal repair (Martinez-Moreno et al., 2018). Therefore, CD133^+^CD34^+^ cell-based therapy may be a promising mechanism to increase the availability of retinal neuroprotective factors with radioprotective effects.

Since both CD133^+^ and CD34^+^ cells have been successfully applied in cell therapies for retinopathy, CD133^+^CD34^+^ cells were directly applied in the present study (Kanji et al., 2014; Fadini et al., 2017; Rong et al., 2018). Further study is needed to investigate the different efficacy between CD133^+^CD34^+^ cells and CD133^+^ or CD34^+^ cells. Despite this limitation, this study suggested CD133^+^CD34^+^ cell transplantation is a promising strategy to treat RR. In our study, we demonstrated the therapeutic effect of *hUCB*-CD133^+^CD34^+^ cells in X-irradiated rats of early stage, in which retinal vascular endothelial cells were mainly injured. In our future studies, we will further explore the optimal therapeutic time window of *hUCB*-CD133^*+*^CD34^*+*^ cells at several time points after X-irradiation. Especially in a later stage with vascular leakage, the effect of *hUCB*-CD133^*+*^CD34^*+*^ cells on vascular leakage should be evaluated. Additionally, the protective effects of transplanted CD133^+^CD34^+^ cells might not be sufficient to attenuate irradiation-induced retinal degeneration for a longer time. As the cell fate of the grafted CD133^+^CD34^+^ cell is determined by the microenvironment, providing a proper microenvironment is essential for the survival, differentiation and integration of these cells during the retinal repair (Kim et al., 2012; Zou et al., 2019). Transplanted CD133^+^CD34^+^ cells exerted a paracrine effect probably by secreting protective factors in our study. But, whether the protective effects of CD133^+^CD34^+^ cells on irradiated retinas result from the differentiation of CD133^+^CD34^+^ cells to replace degenerated or dead neurons is still unknown.

## Conclusion

This is the first study on the radioprotective efficacy of *hUCB*-CD133^+^CD34^+^ cells, a subpopulation of hemopoietic stem cells, on irradiated rat retinas. We demonstrated that intravitreal injection of CD133^+^CD34^+^ cells ameliorated retinal vasculature damage and resulted in visual functional preservation. Further studies should extend these preclinical investigations and explore clinical applications, which may prove beneficial in the near future.

## Data Availability

The raw data supporting the conclusions of this article will be made available by the authors, without undue reservation.

## Abbreviations

CVL, choroidal vascular layer; ERG, Electroretinogram; FGF, Fibroblast growth factor; GCL, Ganglion cell layer; GDNF, glial cell line-derived neurotrophic factor; GM-CSF, Granulocyte-macrophage colony-stimulating factor; HBEGF, heparin-binding epidermal growth factor; hUCB, human umbilical cord blood; hRECs, human retinal microvascular endothelial cells; hNSCs, human neural stem cells; HSCs, Hematopoietic stem cells; IGFBP-1, Insulin-like growth factor binding protein 1; IL-8, Interleukin 8; IL-1β, Interleukin 1β; INL, Inner nuclear layer; MCP-1, Monocyte chemoattractant protein-1; MMP8, Matrix metalloproteinase 8; MIP1α, Macrophage inflammatory protein 1α; ONL, Outer nuclear layer; OP, Oscillating potential; PF4, Platelet factor 4; PHNR, Photopic negative response; RGCs, Retinal ganglion cells; RPE, Retinal pigment epithelium; RR, Radiation retinopathy; TIPM-1, Tissue inhibitors of metalloproteinase 1; WB, Western blot.
