# Supplementary figures and images for: Human Umbilical Cord Blood-Derived CD133+CD34+ Cells Protect Retinal Endothelial Cells and Ganglion Cells in X-Irradiated Rats through Angioprotective and Neurotrophic Factors
Source: Front Cell Dev Biol. 2022 Feb 10;10:801302. doi: 10.3389/fcell.2022.801302 (PMC8866877; doi:10.3389/fcell.2022.801302)

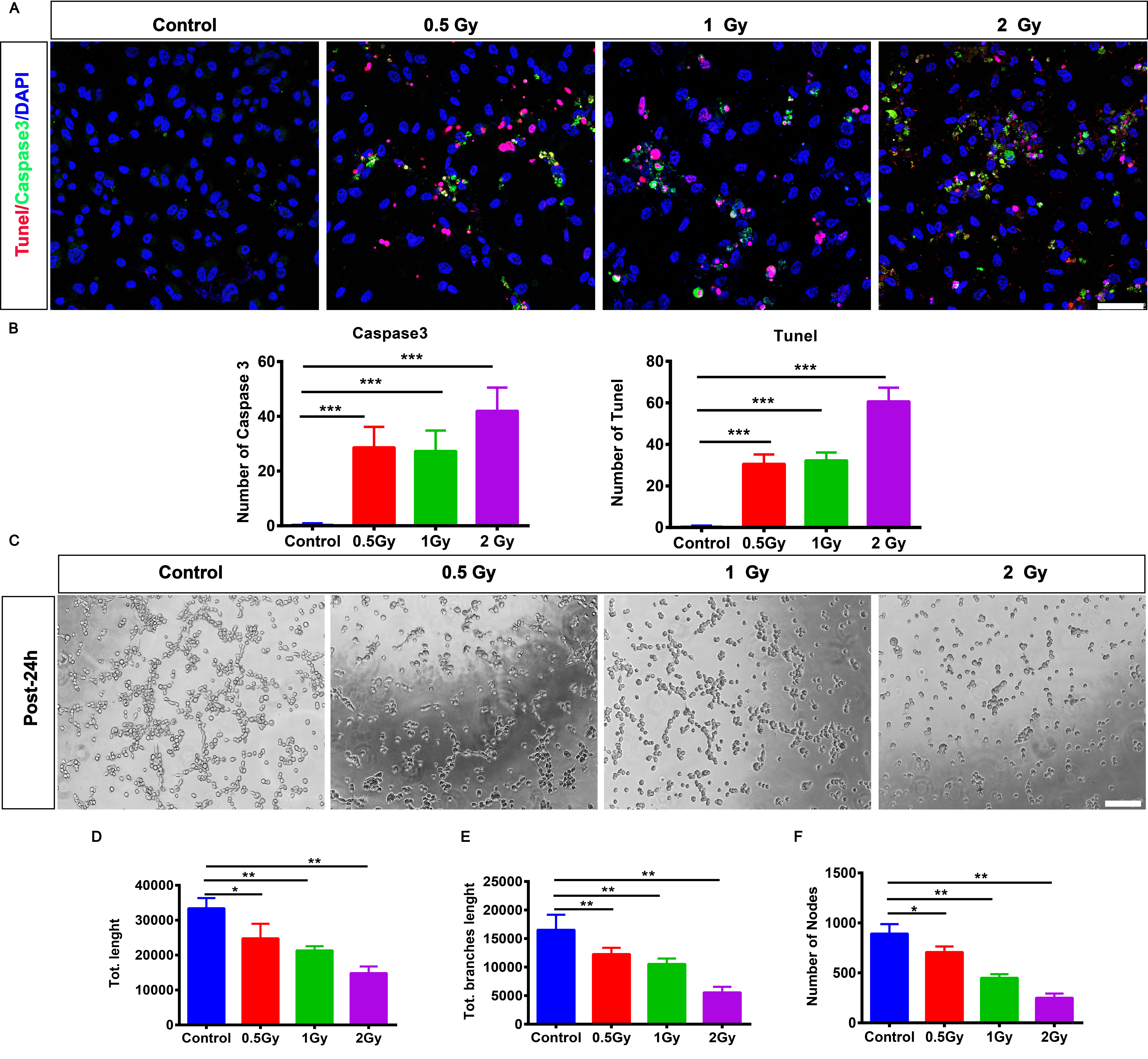

Supplement: Supplementary file 1 [file Image5.jpg]

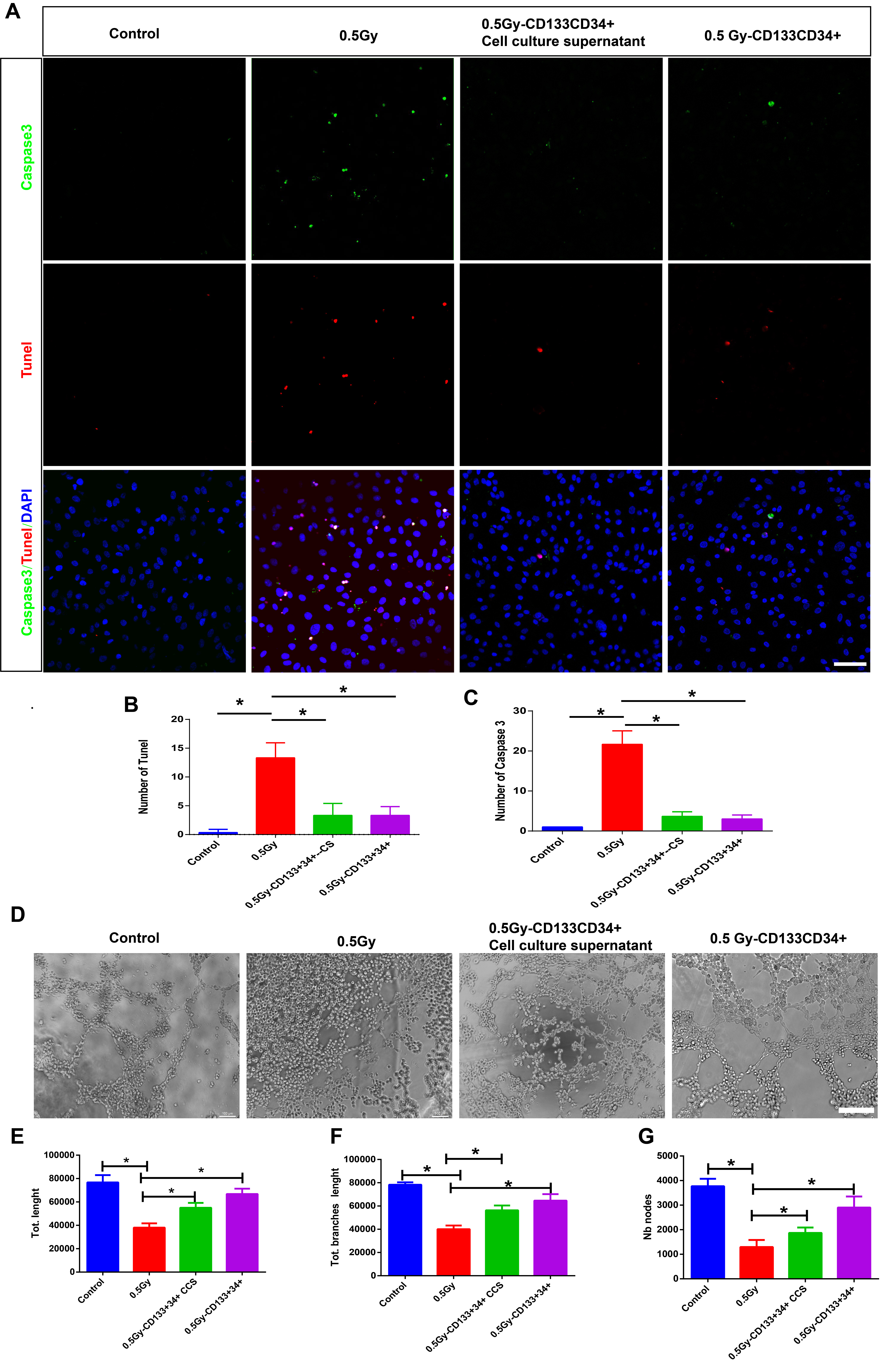

Supplement: Supplementary file 2 [file Image6.jpg]

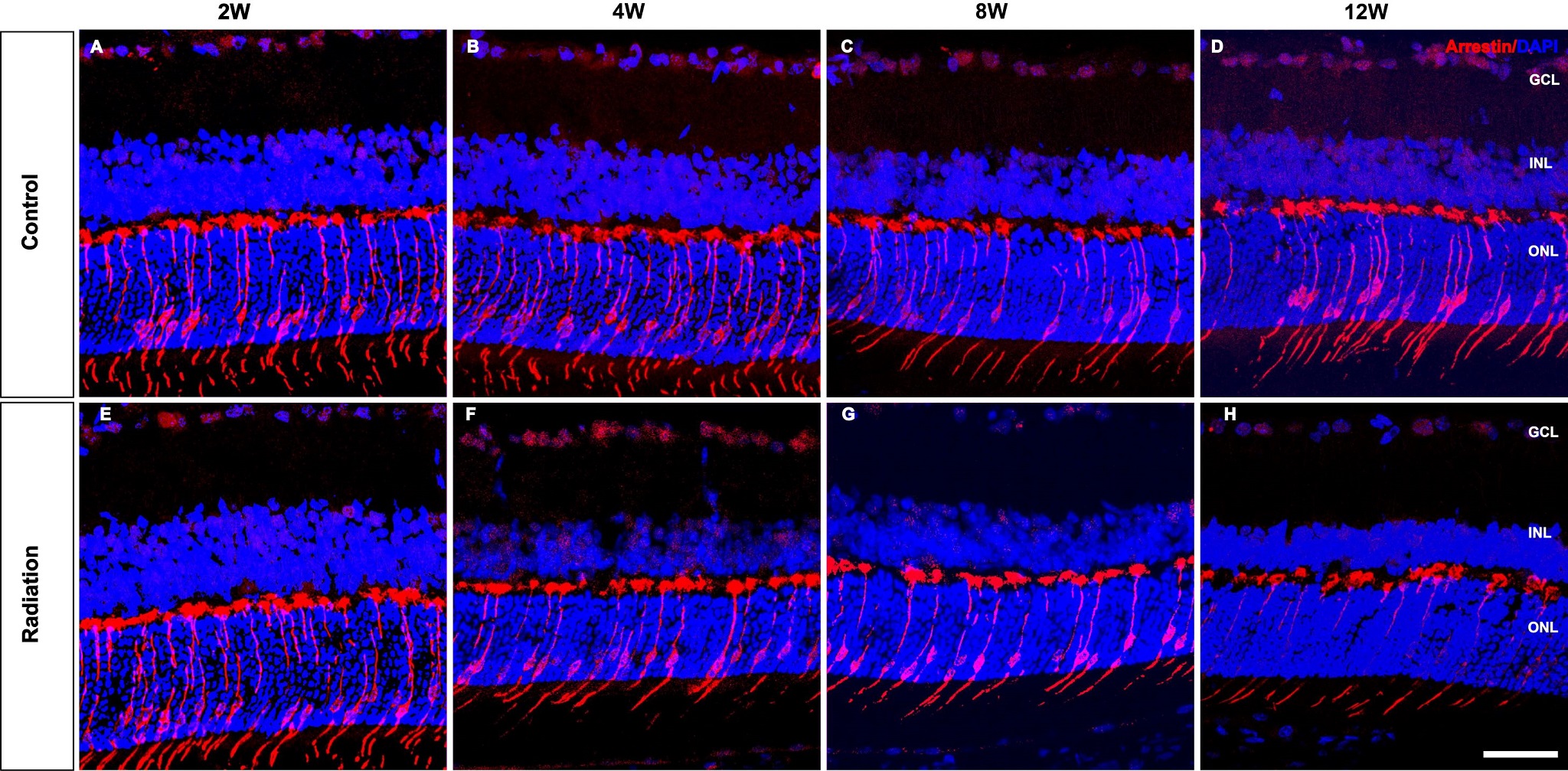

Supplement: Supplementary file 3 [file Image3.jpg]

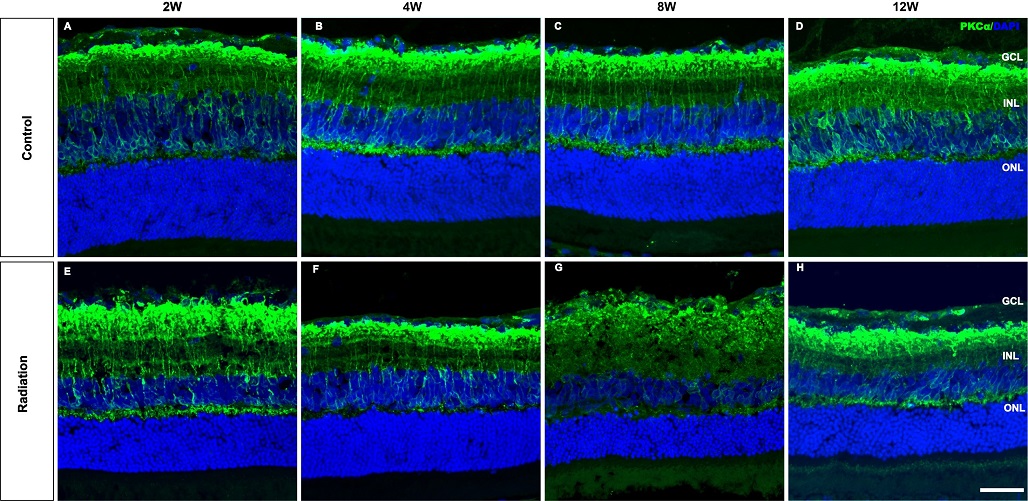

Supplement: Supplementary file 4 [file Image2.jpg]

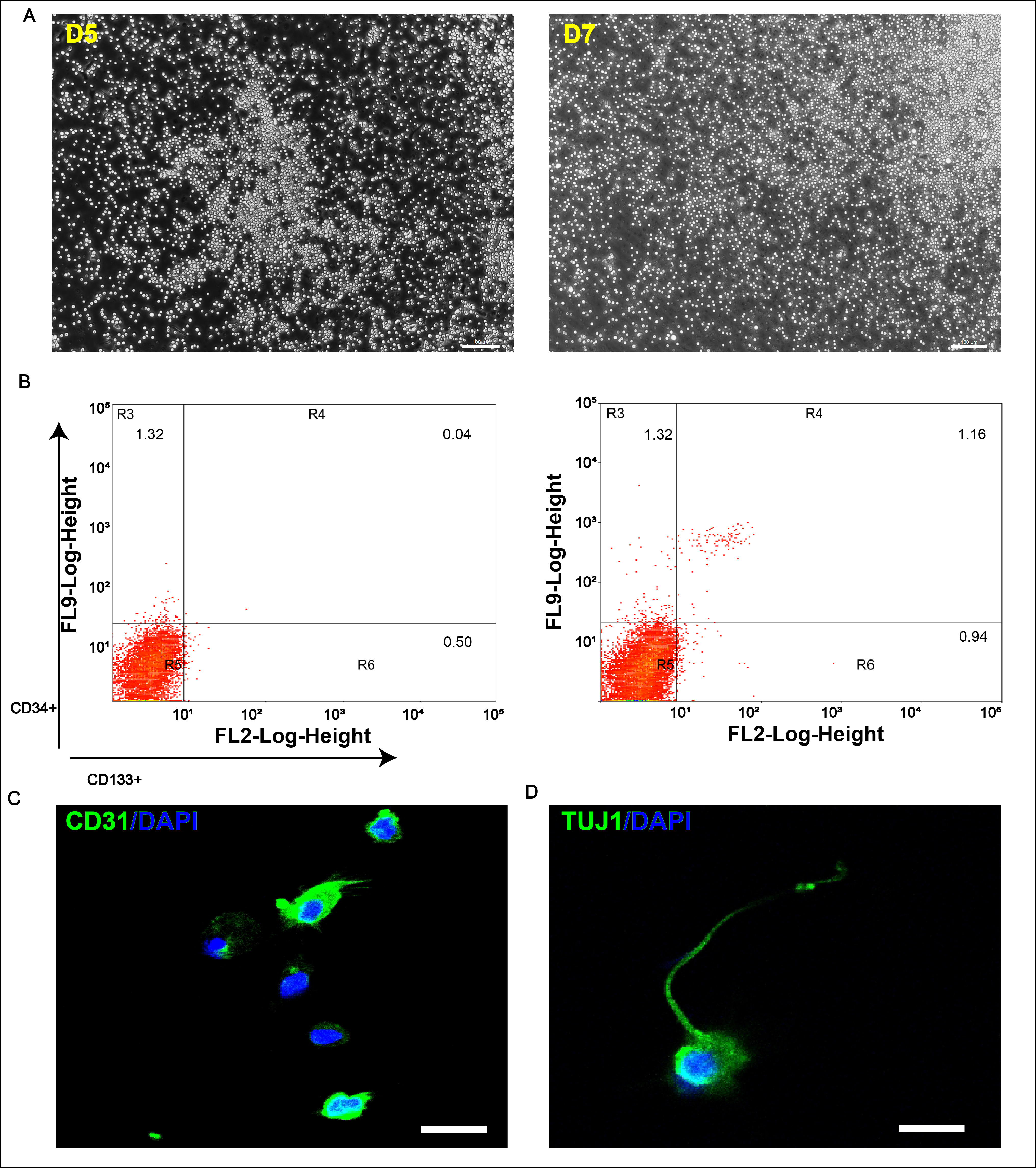

Supplement: Supplementary file 5 [file Image4.jpg]

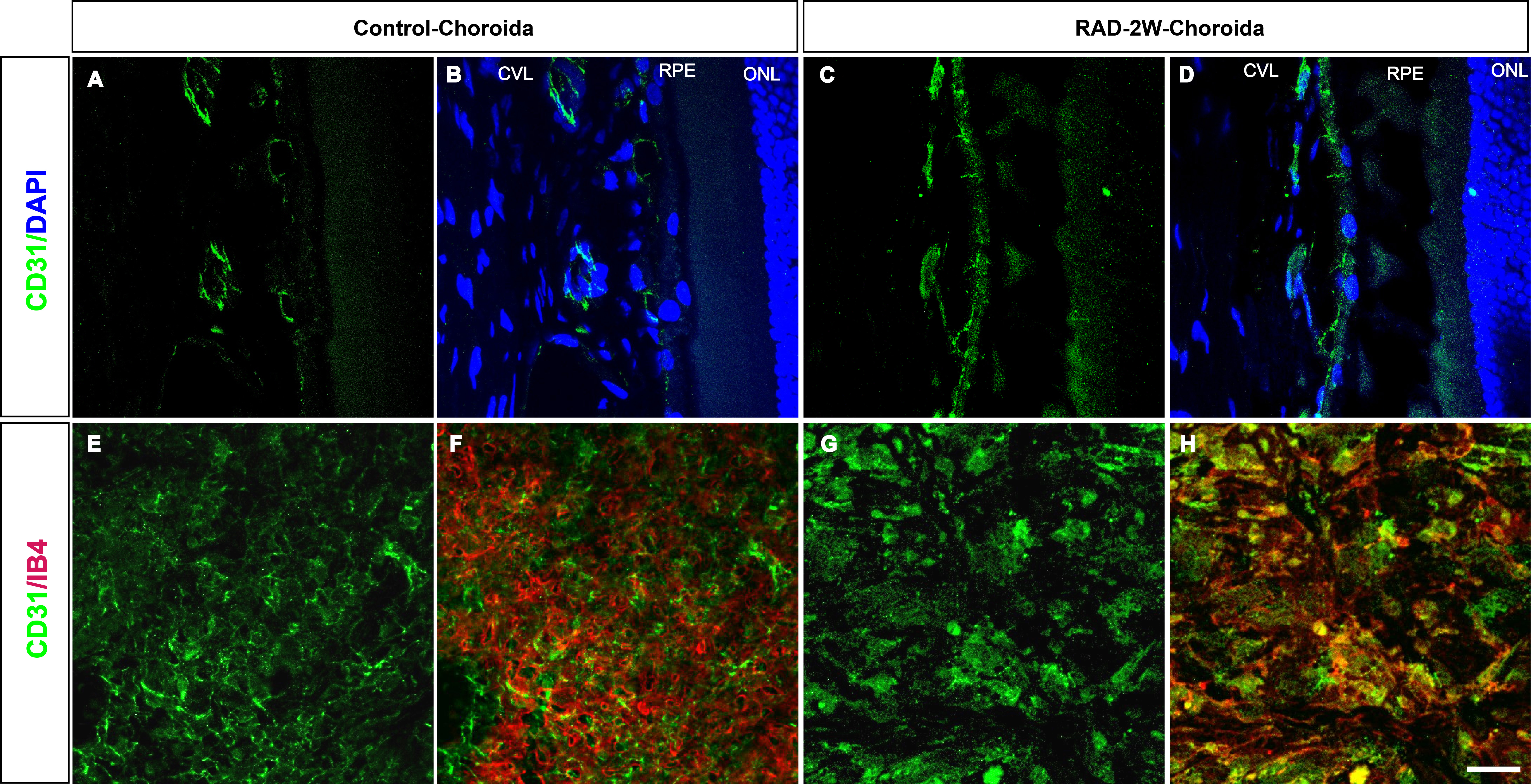

Supplement: Supplementary file 6 [file Image1.jpg]
